# Optimized potassium application rate increases foxtail millet grain yield by improving photosynthetic carbohydrate metabolism

**DOI:** 10.3389/fpls.2022.1044065

**Published:** 2022-11-30

**Authors:** Yanfen Li, Meiqiang Yin, Lulu Li, Jungang Zheng, Xiangyang Yuan, Yinyuan Wen

**Affiliations:** College of Agriculture, Shanxi Agricultural University, Taigu, China

**Keywords:** foxtail millet, potassium fertilization, photosynthetic characteristics, carbohydrate metabolism, yield

## Abstract

Photosynthetic carbohydrate metabolism is an important biochemical process related to crop production and quality traits. Potassium (K) critically contributes to the process of photosynthetic carbon assimilation and carbohydrate metabolism. We explored the effects of potassium fertilization on physiological mechanisms including carbohydrate metabolism in foxtail millet and its yield. Field experiments were performed using two foxtail millet (*Setaria italica* L.) cultivars: 1) Jingu 21 (low-K sensitive); and 2) Zhangza 10 (low-K tolerant). Effect of five different potassium fertilizer (K_2_O) rates (0, 60, 120, 180, and 240 kg·hm^−2^) were tested in two consecutive years, 2020 and 2021. We found that potassium application significantly increases the K content, dry matter accumulation and yield. Jingu 21 and Zhangza 10 had maximum yields at 180 kg·hm^−2^ K application, which were 29.91% and 31.51% larger than without K application, respectively. Excessive K application (K_240_) did not further improve their yields. The suitable K fertilizer application of Jingu 21 and Zhangza 10 are 195.25–204.27 and 173.95–175.87 kg·K2O·hm^−2^, respectively. The net photosynthetic rate (P_n_), ribulose-1,5-bisphosphate carboxylase-oxygenase (Rubisco), phosphoenolpyruvate carboxylase (PEPC), and fructose-1,6-bisphosphatase (FBPase) were positively correlated with the potassium content. Potassium application improved the availability of carbon sources for carbohydrate synthesis. Compared with the K_0_ treatment, variations in the activities of sucrose phosphate synthase (SPS) and sucrose synthase (SuSy) in potassium-treated Jingu 21 (K_60_, K_120_, K_180_, and K_240_) were 17.94%–89.93% and 22.48%–182.10%, respectively, which were greater than those of Zhangza 10 (11.34%–71.12% and 16.18%–109.13%, respectively) and indicate that Jingu 21 is more sensitive to potassium application. The sucrose contents in the Jingu 21 and Zhangza 10 grains were 0.97%–1.15% and 1.04%–1.23%, respectively. The starch contents were 28.99%–37.75% and 24.81%–34.62%, respectively. The sucrose: ratio of Jingu 21 was smaller than that of Zhangza 10, indicating that Jingu 21 utilized nutrients better than Zhangza 10. Stepwise regression and path analysis showed that leaf and grain SuSy activity, by coordinating the source-sink relationship, have the greatest direct effect on Jingu 21 yield, whereas leaf SuSy activity, by promoting the generation of photosynthates at the source leaf, plays a leading role in Zhangza 10 yield increase. In conclusion, optimized K application can increase foxtail millet grain yield by improving photosynthesis and promoting carbohydrate accumulation and distribution.

## Introduction

Millet is one of the main multigrain crops grown in the Loess Plateau region of China, and has advantages of drought resistance, barren tolerance, and a wide adaptability. As multiple cropping indices have increased, the available potassium in the soil of some northern regions can no longer meet the requirements for crop growth and development (DU et al., 2019). To achieve high yields and quality during cultivation, the application of exogenous potassium is indispensable. Cereal crops have high K demands, and the amount of K required for 100 kg of grain is 2–3 kg (Xie et al., 2009). Ye et al. (2020) used the linear plus platform model to determine that the suitable potassium application rate for high rice quality and yield was kg·hm^−2^ of K_2_O. Sun et al. (2014) suggested that 133.5 kg·hm^−2^ of K_2_O was suitable for millet in central Shanxi. Zhang et al. (2014) found that 300 kg·hm^−2^ of N and 300 kg·hm^−2^ of K were the best fertilizer application rates for high yields and efficiencies of Zhangza 5.

Potassium acts both as a nutrient and a signaling agent regulating various metabolic processes in crop growth and development; it plays a vital role in optimizing photosynthetic performance, photosynthate transport, and enzyme activation (Mirza et al., 2018; Xu et al., 2022). Yield is determined mainly by the dry matter accumulation in the stem and sheath before the heading stage, and after, it is determined by leaf photosynthesis (Inoue et al., 2004). In cereal crops, such as rice and millet, organs such as grains act as sinks, and leaves and stems act as sources. Carbohydrates, the most important grain-filling assimilate in rice, are produced by photosynthesis and are mainly transported to grains in the form of soluble sugars (Pan et al., 2011). Potassium can regulate the allocation of carbohydrates between the source and sink organs of crops. Appropriate potassium application not only increases the supply of photosynthates at the source end but also improves sink strength and promotes yield. Potassium deficiency is not only detrimental to photosynthesis but also affects the yield and quality by increasing specific leaf weight and accumulation of soluble sugar in leaves, thereby affecting carbohydrates transport to the sink (Asif et al., 2017). In rice and wheat, appropriate potassium fertilization can: a) promote the transport of soluble sugars and free amino acids from leaves and stems to grains, b) provide sufficient substrates for starch synthesis in grains, c) maintain a high grain-filling rate, and d) improve grain weight and yield (Wang et al., 2003; Hou et al., 2019).

Many important enzymes are involved in carbohydrate synthesis, accumulation, and distribution such as: 1) ribulose-1,5-bisphosphate carboxylase-oxygenase (Rubisco) and phosphoenolpyruvate carboxylase (PEPC), which assimilate CO_2_ into organic carbon (Lian et al., 2021; Siddiqui et al., 2021); 2) fructose-1,6-bisphosphatase (FBPase), which catalyzes the first committed step in the sucrose synthesis pathway and promotes sucrose synthesis by sucrose synthase (SuSy) and sucrose phosphate synthase (SPS) (Ju et al., 2021); 3) acid invertase (AI) and neutral invertase (NI), which control sucrose degradation; and 4) ADPG pyrophosphorylase, which is a rate-limiting enzyme in starch synthesis. Metabolism of carbohydrates (such as sucrose and starch) and the yield are significantly correlated with the activity of these enzymes, which in turn are affected by potassium status. In rice, insufficient absorption of potassium leads to a disorder in glucose metabolism and affects vegetative and reproductive growth (Chen et al., 2018). Potassium deficiency decreases Rubisco activity in cotton leaves (Ali et al., 2018) and PEPC activity in rice (Balkos et al., 2010). However, potassium deficiency does not affect the Rubisco activity and content in ‘Tiefeng 40’, a soybean variety (Wang et al., 2015). In tubers, SuSy activity (decomposition direction) decreased, and AI activity increased with potassium deficiency; the AI activity was positively correlated with the degree of potassium deficiency stress (Liu et al., 2013). In cotton leaves, sufficient potassium supply increases SPS and SuSy activity while decreasing the AI activity (Zahoor et al., 2017). Thus, appropriate potassium application significantly affects carbohydrate accumulation and distribution in crops *via* modulating the activities of carbon metabolism-related enzymes, ultimately affecting the yield.

Millets require potassium in large amounts. Potassium deficiency decreases yield of millets; the yield decrease is attributable to reduced photosynthetic capacity and dry matter accumulation (Cao et al., 2019). However, there are few reports on potassium mediated regulation of various physiological mechanisms such as accumulation, transport, and distribution of carbohydrate during the critical growth stages of millet. Here, the optimal potassium fertilization rates for foxtail millet and the role of potassium in physiological mechanisms regulating carbohydrate accumulation, transport, and distribution were determined by analyzing its effect on the dry matter accumulation, carbon metabolism-enzymes activities, carbohydrate accumulation, and yield. Thus, we provide a theoretical and practical basis for green and efficient millet cultivation.

## Materials and methods

### Experimental conditions

**Figure 1:**
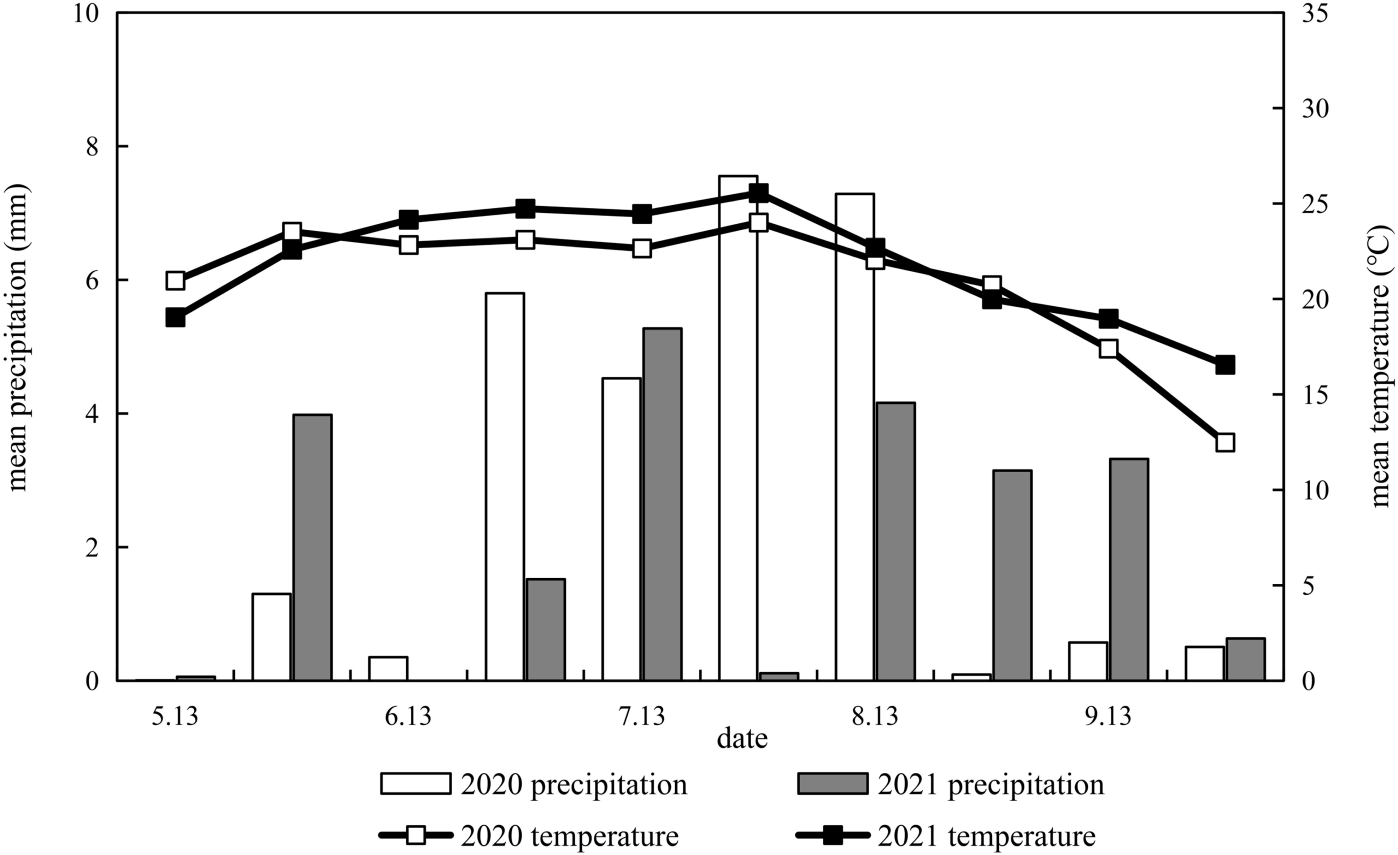
Dynamics of precipitation and temperature during millet growing seasons in 2020 and 2021.

Field experiments were conducted from May to October 2020 and from May to October 2021 at the Shanxi Agricultural University experimental station (Shenfeng Village, Taigu County, Jinzhong City, Shanxi Province, China). From May 13 to October 9 for both years, the average temperature and rainfall were calculated every 15 days, as shown in **Figure 1**. It was sown on May 13, 2020 and harvested on October 6, 2020, and sown on May 18, 2021 and harvested on October 3, 2021. The average temperature during the whole growth period of millet was 21.3°C and 22.2°C, and average rainfall was 419.1 mm and 331.8 mm, in 2020 and 2021, respectively. The rainfall from September 15 to October 9, 2021 (harvest) accounts for 17.4% of the total rainfall received by the crops.

### Experimental design

The experiment was performed using a completely randomized design with three replicates. The soil pH 7.63, organic matter content 16.93 g·kg^−1^, total nitrogen 0.81 g·kg^−1^, alkali-hydrolyzed nitrogen 51.84 mg·kg^−1^, available potassium 98.75 mg·kg^−1^ and available phosphorus 22.37 mg·kg^−1^ in the 0-20 cm soil layer of the experimental field. The applications consisted of five potassium (K_2_O) fertilization rates: 0 (K_0_) as control, 60 (K_60_), 120 (K_120_), 180 (K_180_), and 240 (K_240_) kg·hm^−2^ of K_2_O. Nitrogen (150 kg·hm^−2^ of N) and phosphorus (90 kg·hm^−2^ of P_2_O_5_) fertilizers were applied, of which the N, P, and K were urea (42% N), superphosphate (12% P_2_O_5_), and potassium sulfate (50% K_2_O). Two foxtail millet cultivars, Jingu 21 (low-K sensitive) and Zhangza 10 (low-K tolerant), were used. Square plots, 25 m^2^ in size, were used. For Jingu 21, 0.35 m row-separation and 0.08 m plant-separation was adopted, whereas for Zhangza 10, 0.35 m row-separation and 0.12 m plant-separation was adopted. Local production recommendations were used in management of crops in the field.

### Sampling and processing

Six representative plants were sampled from each plot at the booting and filling stages of millet. The plant height and stem diameter were also measured. The plant samples were then separated into different parts, including the roots, stems, leaves, and panicles. All samples were oven-dried at 105°C for 30 min and then to a constant weight at 85°C to determine the dry weight.

At the mature stage of millet, six representative plants from each plot were harvested for measurement of millet biomass accumulation and partitioning in stems, leaves, and spikes. Panicles were used to measure yield and yield components: parameters such as panicle number, spikelet number, panicle grain weight, and thousand-grain weight.

### Photosynthetic parameters

Photosynthetic parameters were measured at the booting and filling stages in three representative plants from each plot. The functional millet leaves (inverted second leaves) were measured using a photosynthesis system CI-340. After measurement, all leaves were tagged to help sample them again for enzyme extraction and carbohydrate analysis. Photosynthetic parameters were measured from 9:30 to 11:00 A.M. under clear weather at 30 ± 2 °C leaf temperature, 400 ± 50 μmol·mol^−1^ CO_2_, and 1000 ± 50 μmol·m^−2^·s^−1^ photosynthetic photon flux density.

### Enzyme extraction and analysis

The tagged booting stage panicles, leaves, and grains in the mid-filling stage and the corresponding panicle leaves were collected and immediately placed in liquid nitrogen and stored in an ultra-low temperature freezer (–80°C) until enzymatic measurement (each reported index value is the mean value of three repetitions).

The frozen sample (0.2 g) was ground using a chilled mortar containing 2 mL pre-cooled extraction buffer Tris-HCl (pH 8.2) and ground into a slurry in an ice bath. The slurry was centrifuged at 15000 ×*g* at 4°C for 20 min. The supernatant was a crude enzyme solution, which was used for initial Rubisco and PEPC activity assays.

Rubisco was assayed in a 3-mL reaction mixture (1.4 mL of 100 mmol·L^−1^ Tris-HCl (pH 7.8), 0.2 mL of 5 mmol·L^−1^ Adenosine Triphosphate (ATP), 0.2 mL of 0.2 mmol·L^−1^ NaHCO_3_, 0.2 mL of 5 mmol·L^−1^ Nicotinamide adenine dinucleotide (NADH), 0.1 mL of 160 U·L^−1^ phosphocreatine kinase, 0.1 mL of 160 U·L^−1^ glyceraldehyde phosphate dehydrogenase, 0.2 mL of 50 mmol·L^−1^ creatine phosphate, 0.1 mL of 160 U·L^−1^ phosphoglycerate kinase, 0.3 mL of distilled water, and 0.1 mL of crude enzyme solution). The reaction was activated by adding 0.1 mL 10 mmol·L^−1^ Ribulose 1,5-bisphosphate (RuBP). Rubisco activity was measured using a UV-visible spectrophotometer (UV-1800), and the absorbance was measured at 340 nm.

PEPC was assayed in a total volume of 3 mL reaction mixture (1.0 mL 100 mmol·L^−1^ Tris-HCl (pH 8.2), 0.1 mL 5 mmol·L^−1^ nicotinamide adenine dinucleotide phosphate (NADP), 0.1 mL 10.5U Malate dehydrogenase (MDH), 0.1 mL 100 mmol·L^−1^ NaHCO_3_, 1.5 mL distilled water, and 0.1 mL crude enzyme solution). The reaction was activated by adding 0.1 mL 40 mmol·L^−1^ phosphoenolpyruvate (PEP). PEPC activity was measured at an 340 nm.

FBPase was extracted and assayed according to the modified method of Hu et al. (2015).

The enzymes SPS, SuSy, AI, and NI were extracted and assayed according to the modified method of Yang et al. (2017).

The enzyme ADPG pyrophosphorylase activity was measured using a glucose diphosphate pyrophosphorylase activity detection kit (Beijing Solarbio Company).

### Carbohydrate analysis

Dried leaf and grain tissues were ground and passed through a 0.15-mm sieve. The powdered sample (0.2 g) was placed in 6 mL 80% (v/v) ethanol and then incubated in a water bath at 80°C for 30 min. Then, the mixture was centrifuged at 4000× *g* at 4°C for 5 min, the supernatant was collected, 6 mL of 80% ethanol was added to the residue, and the extraction was repeated twice. The three supernatants were collected and diluted to 25 mL using 80% ethanol. The resulting extract was used to determine the sucrose and soluble sugar content by spectrophotometry at OD_480_ and OD_620_, respectively.

The ethanol-insoluble residue was then stored for starch extraction. The starch in the residue was released by boiling with 2 mL of distilled water for 15 min and cooling to 10°C. Then, the starch was hydrolyzed with 9.2 mol·L^−1^ HClO_4_ (2 mL) for 15 min. Distilled water (4 mL) was added to the samples, which were centrifuged at 4000 ×*g* and 4°C for 10 min. The residue was extracted again using 4.6 mol·L^−1^ HClO_4_ (2 mL). The supernatants were combined and diluted with distilled water to 25 mL to obtain starch extract. The starch content was measured using a spectrophotometer at OD_620_.

### Plant potassium content

The powdered sample was digested using a mixture of concentrated H_2_SO_4_ and H_2_O_2_. The potassium content was determined by atomic absorption spectrometry after dilution. Each treatment was repeated thrice.

### Statistical analysis

All figures were drawn using Excel 2010, all data were subjected to an analysis of variance with SPSS 25.0, and the difference between mean values was determined using the least significant difference (*P* = 0.05) test. Stepwise regression, path analyses and linear plus platform model fitting were performed using SPSS 25.0. Stepwise regression and path analyses was performed to clarify the effect of carbohydrate metabolism enzyme activity on yield.

## Results

### K Content in millet leaf and grains increases with potassium application

The K content of millet leaves and grains was considerably affected by potassium application (**Table 1**). The K contents of the leaves and grains increased under K application to different degrees than those under K_0_ treatment. Compared with K_0_ treatment, the K contents of the leaves and grains of both cultivars under K_60_ treatment did not increase significantly (except for the K contents of Zhangza 10 leaves in the filling stage in 2021). The K contents reached their maximum levels under the K_180_ treatment, and no significant difference was observed between the K_120_ and K_180_ treatments. Excessive K application (K_240_) did not increase the K contents of the crops continuously. Compared with the K_0_ treatment, the K contents of the Jingu 21 leaves in the booting and filling stages increased by 26.15% and 29.61%, respectively, whereas the K content of the grains increased by 9.10% under the K_180_ treatment in 2020. Similar results were obtained for 2021: leaf K contents increased by 24.35% and 34.51% in the booting and filling stages, respectively, and grain K contents increased by 22.79% in the filling stage. The increases in the K contents of the Zhangza 10 leaves in the booting stage, leaves in the filling stage, and grains in the filling stage were 38.30%, 21.72%, and 23.08% in 2020, and 22.01%, 38.43%, and 34.43% in 2021, respectively.

**Table 1:** Effect of potassium application on K content in foxtail millet leaves and grains.

Cultivar	K rates kg·hm^−2^	2020			2021		
		Booting stage	Filling stage		Booting stage	Filling stage	
		Leaf (%)	Leaf (%)	Grain (%)	Leaf (%)	Leaf (%)	Grain (%)
JG21	K_0_	0.94 ± 0.05b	0.69 ± 0.04b	0.29 ± 0.02b	0.89 ± 0.07b	0.57 ± 0.09c	0.19 ± 0.01d
	K_60_	0.97 ± 0.08b	0.73 ± 0.09b	0.30 ± 0.02ab	0.94 ± 0.04b	0.61 ± 0.06bc	0.20 ± 0.01cd
	K_120_	1.17 ± 0.10a	0.79 ± 0.07ab	0.32 ± 0.03ab	1.11 ± 0.07a	0.69 ± 0.04abc	0.27 ± 0.02a
	K_180_	1.19 ± 0.11a	0.89 ± 0.09a	0.32 ± 0.02ab	1.11 ± 0.05a	0.77 ± 0.08a	0.23 ± 0.01ab
	K_240_	1.08 ± 0.09ab	0.74 ± 0.05b	0.34 ± 0.04a	1.00 ± 0.10ab	0.73 ± 0.06ab	0.22 ± 0.00bc
ZZ10	K_0_	0.94 ± 0.08c	0.74 ± 0.08b	0.26 ± 0.02b	1.03 ± 0.11c	0.54 ± 0.06c	0.20 ± 0.01c
	K_60_	1.10 ± 0.12bc	0.78 ± 0.07ab	0.28 ± 0.04ab	1.05 ± 0.09c	0.69 ± 0.05b	0.23 ± 0.01bc
	K_120_	1.27 ± 0.12ab	0.90 ± 0.07a	0.29 ± 0.03ab	1.31 ± 0.15a	0.74 ± 0.06ab	0.26 ± 0.01ab
	K_180_	1.30 ± 0.08a	0.90 ± 0.07a	0.32 ± 0.03a	1.26 ± 0.04ab	0.75 ± 0.08ab	0.27 ± 0.04a
	K_240_	1.25 ± 0.04ab	0.85 ± 0.07ab	0.30 ± 0.03ab	1.12 ± 0.09bc	0.83 ± 0.08a	0.27 ± 0.01a

Different normal letters within the same column indicate significant differences in responses of the same cultivar to different potassium applications at P< 0.05 using the LAD method. The values shown are mean values ± denotes standard deviation. JG21, Jingu 21; ZZ10, Zhangza 10.

### Net photosynthetic rate increases with potassium application

**Figure 2:**
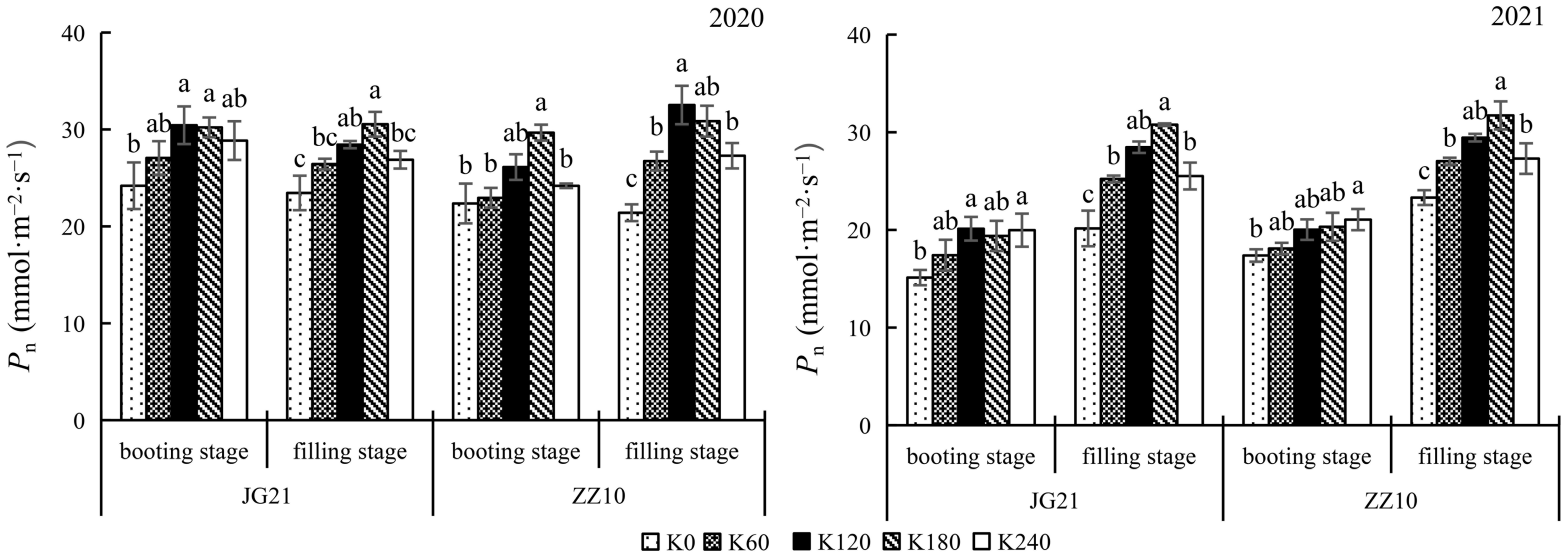
Effects of potassium application rate on net photosynthetic rate of foxtail millet cultivars, Jingu 21 (JG21) and Zhangza 10 (ZZ10). Potassium applications include 5 level 0 (K_0_) as control, 60 (K_60_), 120 (K_120_), 180 (K_180_), and 240 (K_240_) kg·hm^−2^ of K_2_O. The different normal letters indicate significant differences in different treatments of the same variety at the 0.05 level. Values are means ± SE (n = 3).

The net photosynthetic rate (*P*_n_) increased as the millet crops grew leading to leaf expansion and an increase in leaf area. Potassium application increased the *P*_n_ at the booting and filling stages (**Figure 2**). Compared with the control, K_180_ application significantly increased the *P*_n_ in Jingu 21 by 24.90% and 30.23% (2020), and by 28.22% and 52.74% (2021), at the booting and filling stages, respectively. In Zhangza 10, *P_n_* increased by 32.67% and 44.15% (2020), and by 16.89% and 36.11% (2021), at the booting and filling stages, respectively. The *P*_n_ variation range at the filling stage was greater than that at the booting stage.

### Photosynthetic carbon assimilation enzymes activities increased upon potassium application

**Figure 3:**
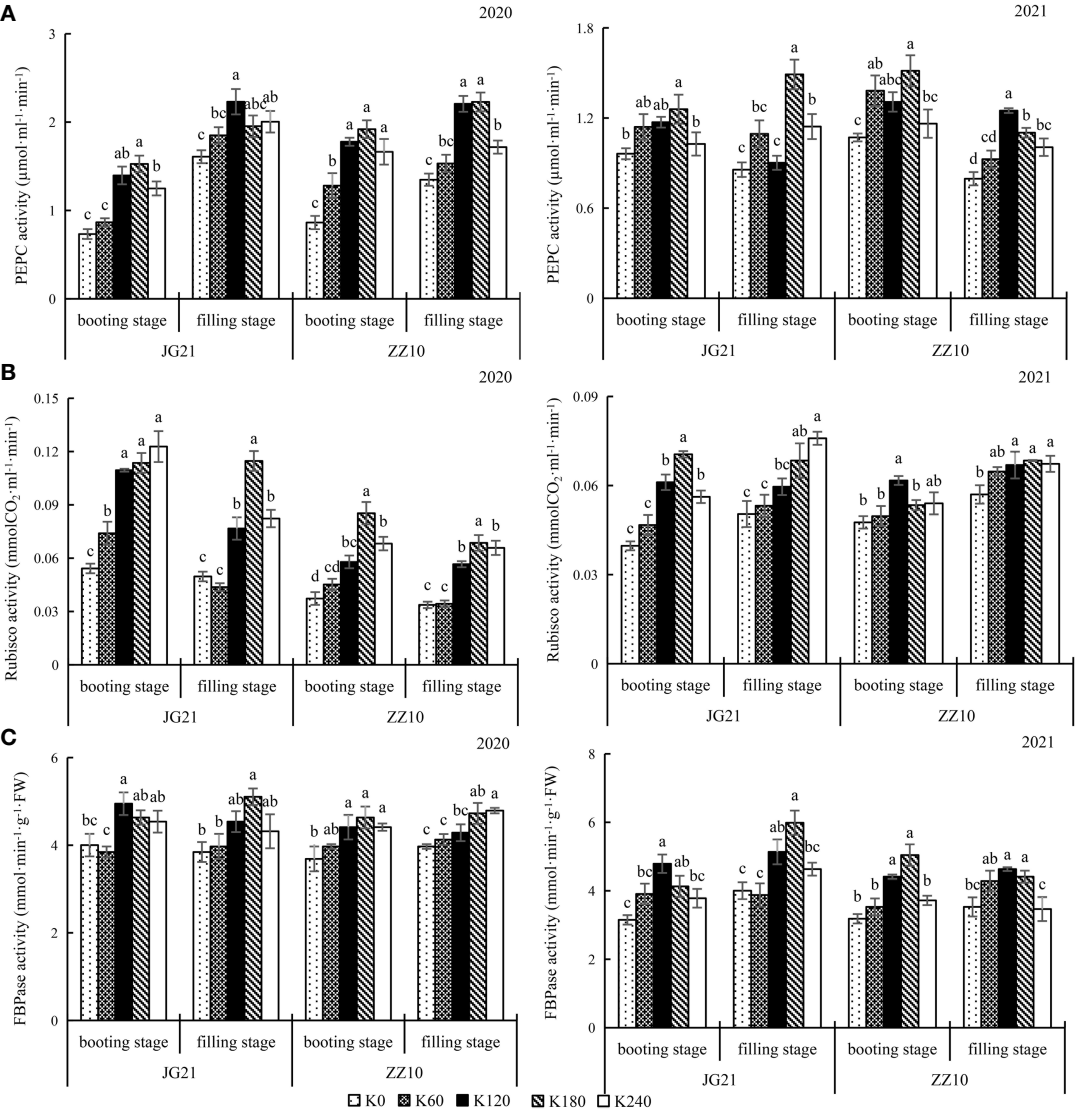
Effects of potassium application rate on key enzymes of carbon assimilation and FBPase of foxtail millet cultivars, Jingu 21 (JG21) and Zhangza 10 (ZZ10). Potassium applications include 5 level 0 (K_0_) as control, 60 (K_60_), 120 (K_120_), 180 (K_180_), and 240 (K_240_) kg·hm^−2^ of K_2_O. The different normal letters indicate significant differences in different treatments of the same variety at the 0.05 level. Values are means ± SE (n = 3). **(A)** PEPC, phosphoenolpyruvate carboxylase. **(B)** Rubisco, ribulose-1,5-bisphosphate carboxylase-oxygenase. **(C)** FBPase, Fructose-1,6-bisphosphatase.

Activities of carbon assimilation enzymes (PEPC and Rubisco) were enhanced by potassium application at the booting and filling stages (**Figure 3**). There were no significant differences in the booting stage photosynthetic parameters with K_120_ and K_180_ applications for both cultivars; the results were similar for the 2020 and 2021 crops. Compared to the control, K_180_ application significantly increased the PEPC activity by 69.53% and 81.90%, in Jingu 21 and Zhangza 10, respectively; particularly, the filling stage PEPC activity significantly increased by 56.39% and 51.89% in Jingu 21 and Zhangza 10, respectively (**Figure 3A**). Compared to the control, K_180_ application significantly increased the booting stage Rubisco activity by 93.57% and 70.44%, in Jingu 21 and Zhangza 10, respectively; the filling stage Rubisco activity significantly increased by 45.02% and 61.78%, in Jingu 21 and Zhangza 10, respectively (**Figure 3B**). FBPase activity changes followed a trend similar to that of PEPC and Rubisco activity. The FBPase activity of the two cultivars was not significantly different between the K_120_ and K_180_ applications, and not significantly different between the K_0_ and K_60_ applications. The average of Jingu 21 FBPase activity increased by 21.98% and 19.62% at the booting and filling stage, respectively; and that of Zhangza 10 was increased by 24.68% and 15.94%, at the booting and filling stages, respectively (**Figure 3C**).

### Potassium application enhances sucrose synthesizing enzymes and diminishes sucrose degrading enzymes

**Figure 4:**
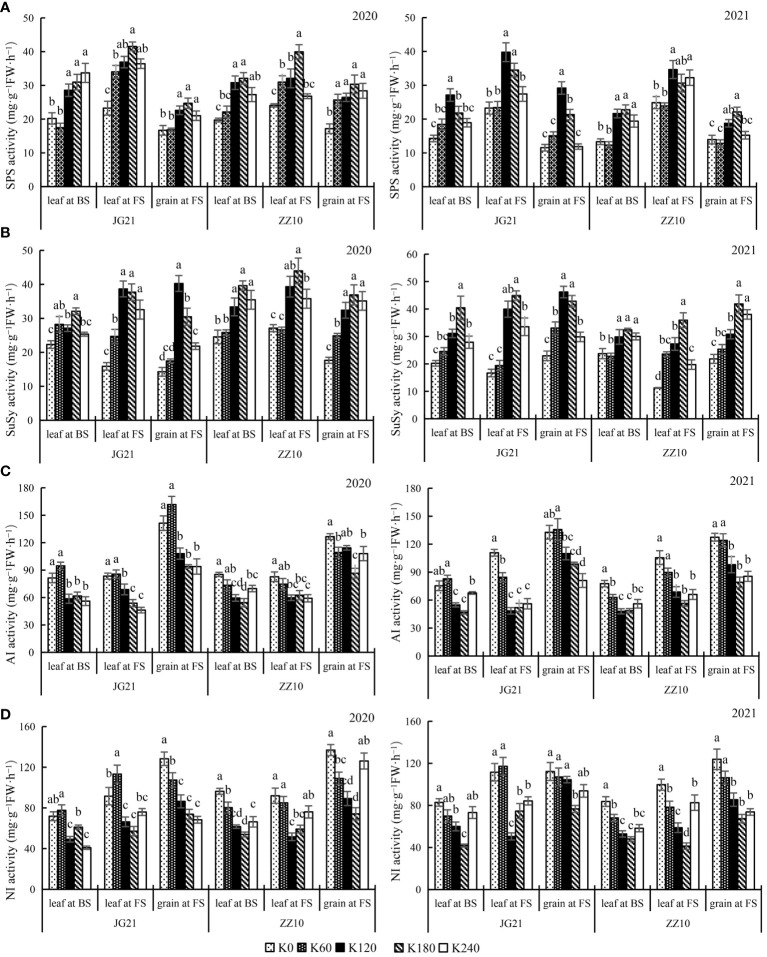
Effects of potassium application rate on sucrose metabolism-related enzyme activities of foxtail millet cultivars, Jingu 21 (JG21) and Zhangza 10 (ZZ10). Potassium applications include 5 level 0 (K_0_) as control, 60 (K_60_), 120 (K_120_), 180 (K_180_), and 240 (K_240_) kg·hm^−2^ of K_2_O. BS: booting stage, FS: filling stage. The different normal letters indicate significant differences in different treatments of the same variety at the 0.05 level. Values are means ± SE (n = 3). **(A)** SPS, sucrose phosphate synthase. **(B)** SuSy, sucrose synthase. **(C)** AI, Acid invertase. **(D)** NI, neutral invertase.

The response of sucrose metabolism-related enzymes to potassium application rates varied with plant parts and the growth stages; however, crops of both the years showed a similar trend (**Figure 4**). SPS and SuSy are enzymes that catalyze sucrose synthesis. SPS and SuSy activities showed a peaking-response to increases in potassium application rates. For both years, compared with the control, under potassium treated (K_60_, K_120_, K_180_ and K_240_), the average variation amplitude of SPS activity in leaves at the booting stage, leaves at the filling stage, and grains at the filling stage in Jingu 21 were 47.41%, 47.25%, and 47.70%, respectively, and those in Zhangza 10 were 42.69%, 29.44%, and 44.53%, respectively (**Figure 4A**). The average changes in SuSy activity in Jingu 21 (at the same stages and organs mentioned above) were 39.92%, 108.46%, and 79.16%, respectively, and those in Zhangza 10 were 29.96%, 86.22%, and 69.55%, respectively (**Figure 4B**). The enzyme activity variation amplitude was smaller in Zhangza 10.

AI and NI catalyze the degradation of sucrose (Wang et al., 2019). Their response to potassium application is converse those of SPS and SuSy, indicating that potassium application results in reduced sucrose decomposition. The AI and NI activities of Jingu 21 and Zhangza 10 peaked at K_0_ or K_60_ application, whereas those in leaves showed an increasing trend as growth progressed. Moreover, AI and NI activities in grains were greater than those in leaves (**Figure 4**). Compared with that in the control, the AI activity of Jingu 21 was significantly decreased by 33.55% (2020) and 25.71% (2021) under K_180_ treatment, and the AI activity of Zhangza 10 was significantly decreased by 31.63% (2020) and 37.95% (2021) under K_180_ treatment (**Figure 4C**). Grain NI activity of Jingu 21 was significantly decreased by 42.50% (2020) and 45.83% (2021), and that of Zhangza 10 was significantly decreased by 31.70% (2020) and 45.59% (2021) (**Figure 4D**).

### Potassium application increases soluble carbohydrate contents in grains at filling stage

**Figure 5:**
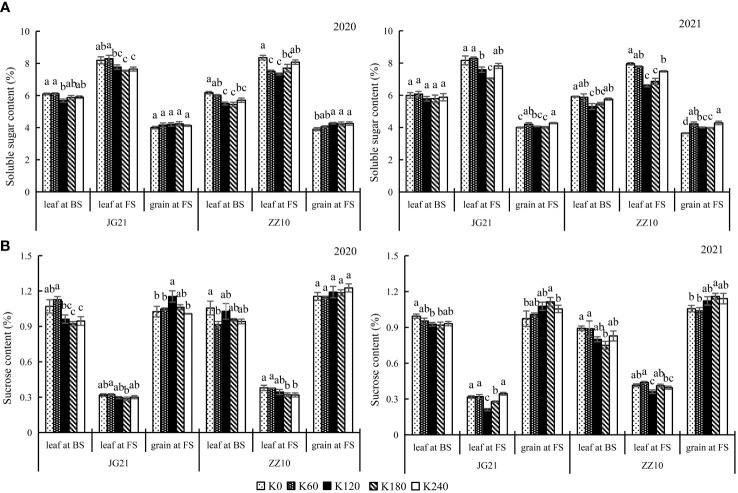
Effects of potassium application rate on soluble sugar and sucrose content of foxtail millet cultivars, Jingu 21 (JG21) and Zhangza 10 (ZZ10). Potassium applications include 5 level 0 (K_0_) as control, 60 (K_60_), 120 (K_120_), 180 (K_180_), and 240 (K_240_) kg·hm^−2^ of K_2_O. BS, booting stage, FS, filling stage. The different normal letters indicate significant differences in different treatments of the same variety at the 0.05 level. Values are means ± SE (n = 3). **(A)** soluble sugar content. **(B)** sucrose content.

Soluble sugar content reflects the carbohydrate supply in plants (Sun et al., 2022). Potassium application significantly affects the soluble sugar and sucrose content in leaves and grains. Here, the changes in soluble sugar and sucrose content in leaves and grains in response to potassium application were inconsistent. While potassium application reduced the soluble sugar and sucrose content in leaves, it increased their levels in grains (**Figures 5A, B**). The soluble sugar content varied as follows: leaves at the filling stage> leaves at the booting stage> grains at the filling stage. The sucrose content varied as follows: grain at the filling stage> leaf at the booting stage> leaf at the filling stage. No significant differences were observed between the soluble sugar and sucrose contents of Jingu 21 leaves in the booting and filling stages under the K_120_ and K_180_ treatments, and no significant differences were observed among the K_120_, K_180_, and K_240_ treatments in the booting stage. There were no significant differences in grain soluble sugar contents among the treatments in 2020 of Jingu 21; however, grain soluble sugar content peaked under the K_240_ treatment in 2021, which was significantly higher (6.85%) than the K_0_ treatment. The Jingu 21 grain sucrose content peaked under the K_120_ treatment in 2020, which was significantly higher (12.56%) than the K_0_ treatment, and peaked under the K_180_ treatment in 2021, which was significantly higher (14.55%) than the K_0_ treatment. No significant differences in grain sucrose contents were observed between 2020 and 2021 under the K_120_ and K_180_ treatments. The leaf soluble sugar content of Zhangza 10 peaked under the K_0_ treatment, which differed significantly from those under the K_120_ and K_180_ treatments. No significant differences in Zhangza 10 grain sucrose contents were observed among the treatments in 2020, and grain sucrose contents peaked under the K_180_ treatment in 2021, which was significantly higher (9.70%) than the K_0_ treatment. Except for the K_180_ treatment, there were no significant differences between the other treatments.

### Optimal potassium application enhances activity of the starch synthesizing enzyme ADPG pyrophosphorylase

**Figure 6:**
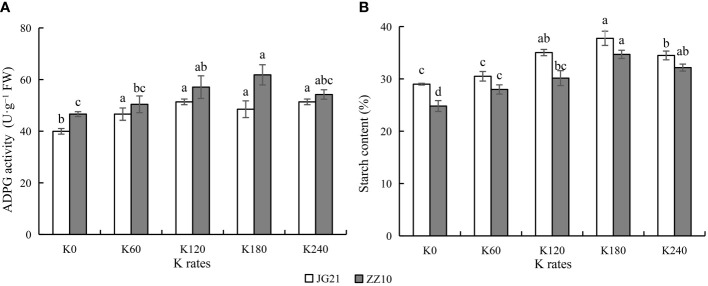
Effects of potassium application rate on ADPG activity and starch content of foxtail millet grains at grain filling stage (2021). Two cultivars were tested, Jingu 21 (JG21) and Zhangza 10 (ZZ10). Potassium applications include 5 level 0 (K_0_) as control, 60 (K_60_), 120 (K_120_), 180 (K_180_), and 240 (K_240_) kg·hm^−2^ of K_2_O. The different normal letters indicate significant differences in different treatments of the same variety at the 0.05 level. Values are means ± SE (n = 3). **(A)** ADPG, ADPG pyrophosphorylase. **(B)** starch content.

ADPG pyrophosphorylase is a rate-limiting enzyme that catalyzes starch synthesis. With increasing potassium application rates, ADPG pyrophosphorylase activity showed a peaking-response. ADPG pyrophosphorylase activity in Jingu 21 peaked at the K_120_ and K_240_ (with a significant increase of 28.57% compared to that in the control). ADPG pyrophosphorylase activity in Zhangza 10 peaked at K_180_ (with a significant increase of 32.65% compared to that in the control) (**Figure 6A**). The starch content was significantly affected by potassium application, peaking at K_180_ application rate, and subsequently decreasing at K_240_ (**Figure 6B**). The ADPG pyrophosphorylase activity changes followed the Zhangza 10> Jingu 21 pattern, whereas the changes in starch content followed the Jingu 21> Zhangza 10 pattern. In the middle stage of grain filling, the sucrose content of Jingu 21 ranged from 0.97%–1.15%, and that of Zhangza 10 ranged from 1.04%–1.23% (**Figure 5B**). The Jingu 21 and Zhangza 10 cultivars had 28.99%–37.75% and 24.81%–34.62% starch contents, respectively. The sucrose/starch ratio of Jingu 21 was less than that of Zhangza 10, and the assimilate utilization ability of Jingu 21 was higher.

### Relationship of *P*_n_, carbohydrates, and carbohydrates metabolizing enzymes with K content

A positive correlation between *P*_n_ and the activities of PEPC, FBPase, SPS, SuSy, and K content was observed in both cultivars (**Table 2**). Further, a negative correlation between the activities of AI and NI, soluble sugar and sucrose, and K contents was observed in both cultivars (**Table 2**). The higher K content in the leaves improves the photosynthetic capacity of millet, by providing more carbon sources for carbohydrate synthesis, better coordination of the source-sink relationship, and preventing accumulation of photosynthates.

**Table 2:** Correlation analysis of leaf K content with *P*_n_, carbohydrates, and carbohydrate metabolizing enzymes.

Cultivar	*P*_n_	PEPC	Rubisco	FBPase	SPS	SuSy	AI	NI	Soluble sugar	Sucrose
JG21	0.577**	0.586**	0.655**	0.399*	0.613**	0.565**	-0.542**	-0.451*	-0.380*	–0.099
ZZ10	0.467**	0.679**	0.201	0.367*	0.440*	0.701**	-0.650**	-0.377*	–0.046	–0.321

* and ** indicate significant difference at 0.05 and 0.01 probability levels, respectively. *P_n_*, net photosynthetic rate; JG21, Jingu 21; ZZ10, Zhangza 10; PEPC, phosphoenolpyruvate carboxylase; Rubisco, ribulose-1,5-bisphosphate carboxylase-oxygenase; FBPase, Fructose-1,6-bisphosphatase; SPS, sucrose phosphate synthase; SuSy, starch synthase; AI, acid invertase; NI, neutral invertase.

### Dry matter accumulation and partitioning in the shoots of millet was enhanced by potassium application

Dry matter accumulation in the shoots of millet in different periods was improved by K application (**Table 3**). During the growth period, dry matter accumulation in the millet shoots increased gradually. With increasing potassium application rates, the stem, leaf, and total weights all increased. In the booting and filling stages, shoot dry matter accumulation peaked under the K_180_ treatment. When the potassium application reached 240 kg·hm^−2^ (K_240_), dry matter accumulation did not increase significantly. Compared with the K_0_ treatment, the total weights of Jingu 21 in the booting and filling stages increased by 44.40% and 15.74% (2020) and by 49.80% and 31.10% (2021), while those of Zhangza 10 increased by 20.55% and 19.37% (2020) and by 13.74% and 25.70% (2021), respectively. Compared with the K_0_ treatment, the K_180_ treatment increased the Jingu 21 grain dry matter by 16.32% (2020) and 56.22% (2021), and that of Zhangza 10 by 19.88% (2020) and 32.87% (2021). Compared with the K_0_ treatment, the potassium application treatments (K_60_, K_120_, K_180_, and K_240_) increased the Jingu 21 grain and total weights by 0.46% (2020) and 2.12% (2021), while those of Zhangza 10 increased by 0.49% (2020) and 0.84% (2021) on average. Potassium application increased grain dry matter accumulation; however, the percentage of grain dry matter distribution did not increase significantly.

**Table 3:** Effect of potassium application on dry matter accumulation in foxtail millet shoot.

Year	Cultivar	K rates kg·hm^−2^	Booting stage			Filling stage			
			Stem (g)	Leaf (g)	Total (g)	Stem (g)	Leaf (g)	Spike (g)	Total (g)
2020	JG21	K_0_	8.54c	5.61c	14.15d	20.21ab	8.80c	20.11c	49.12c
		K_60_	9.69bc	6.53b	16.21c	22.32ab	8.93bc	21.84bc	53.09bc
		K_120_	10.94b	7.05ab	17.98b	23.53ab	10.55a	24.76a	58.84a
		K_180_	12.61a	7.83a	20.44a	23.55ab	9.90ab	23.40ab	56.85ab
		K_240_	10.72b	7.67a	18.93b	24.55a	9.59abc	22.90abc	57.03ab
	ZZ10	K_0_	7.19bc	5.33a	12.52b	10.08bc	5.47c	16.63c	32.18b
		K_60_	6.90c	5.47a	12.37b	9.91c	5.80bc	17.34bc	33.06b
		K_120_	9.26a	5.96a	15.23a	11.34abc	6.59ab	19.56ab	37.49a
		K_180_	9.01a	6.08a	15.09a	11.45ab	7.02a	19.94a	38.41a
		K_240_	8.38ab	5.68a	14.07ab	11.62a	6.42a	20.09a	38.13a
2021	JG21	K_0_	7.84c	4.49c	12.33c	17.46c	7.65b	8.70d	33.81d
		K_60_	8.05c	4.97bc	13.02c	18.93bc	7.85b	8.53d	35.30cd
		K_120_	11.30a	5.94a	17.24a	20.20ab	8.14ab	11.53b	39.87b
		K_180_	12.15a	6.33a	18.48a	22.01a	8.72a	13.58a	44.32a
		K_240_	9.82b	5.30b	15.12b	19.25bc	7.85b	10.14c	37.24bc
	ZZ10	K_0_	8.95b	4.98b	13.93b	11.45c	5.78b	15.47c	32.70c
		K_60_	9.66ab	5.41ab	15.07ab	11.12c	5.97ab	15.36c	32.44c
		K_120_	10.37a	5.48a	15.84a	12.62b	6.43a	17.50b	36.55b
		K_180_	10.17a	5.68a	15.85a	14.10a	6.46a	20.55a	41.10a
		K_240_	9.82ab	5.45a	15.27a	13.04ab	6.33ab	18.50b	37.86b

JG21, Jingu 21; ZZ10, Zhangza 10.

### Potassium application improves yield: Certain yield components are enhanced over others

**Figure 7:**
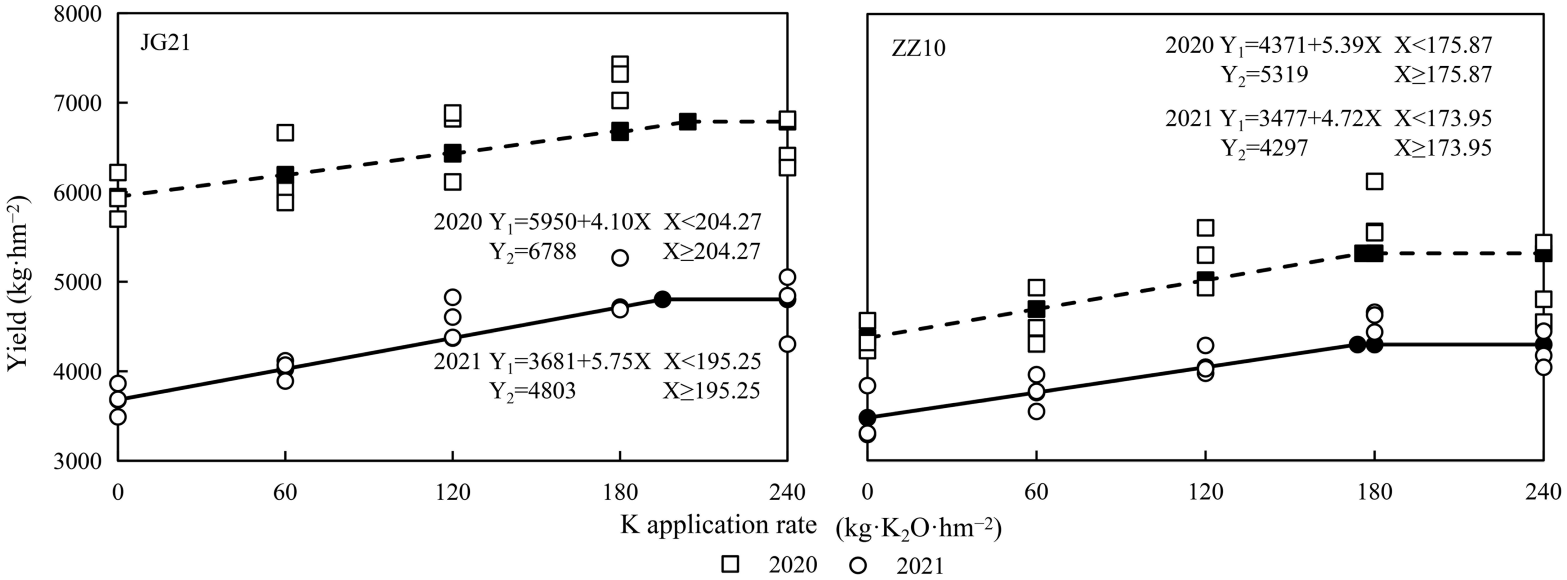
Effect of potassium application rate on yield of millet varieties, Jingu 21 (JG21) and Zhangza 10 (ZZ10).

The millet yields under different K application rates are shown in **Figure 7**. With increasing potassium application rate, the yield initially increased and then stabilized or decreased slightly (**Table 4**). The linear plus platform model was used to fit the effect equations between the potassium application rates and millet yields in 2020 and 2021. The optimal potassium application rates for Jingu 21 and Zhangza 10 were 195.25–204.27 and 173.95–175.87 kg·K_2_O·hm^−2^, respectively. Compared to the control, the Jingu 21 and Zhangza 10 yields increased by 4.03%–37.84% and 4.65%–31.56% in both years, respectively. The variations in the Jingu 21 yield were larger than those of Zhangza 10, indicating that Jingu 21 was more sensitive to potassium fertilizer application.

**Table 4:** Effects of potassium application rate on the yield components of foxtail millet.

V	K rates(kg·hm^−2^)	2020					2021				
		Panicle number(No.·m^-2^)	Spikelet number(No.)	Grain weight(g)	1000 grain weight(g)	Yield(kg·hm^−2^)	Panicle number(No.·m^-2^)	Spikelet number(No.)	Grain weight(g)	1000 grain weight(g)	Yield(kg·hm^−2^)
JG21	K_0_	33.50a	104.33c	22.19b	2.72b	5950.00c	34.17a	104.50a	14.59c	2.70c	3680.58b
	K_60_	35.34a	103.33c	23.38ab	2.81b	6190.00bc	35.34a	106.75a	15.83bc	2.78bc	4025.49b
	K_120_	35.84a	117.67ab	25.23ab	2.87ab	6606.673b	34.84a	111.00a	17.90a	2.89ab	4603.36a
	K_180_	35.67a	121.33a	26.18a	3.03a	7258.33a	35.84a	109.25a	18.22a	2.99a	5073.26a
	K_240_	34.84a	109.67bc	25.59a	2.92ab	6500.00bc	34.84a	106.25a	17.04ab	2.93ab	4733.11a
ZZ10	K_0_	28.50b	112.67a	16.48b	2.81b	4371.67c	25.67a	119.25a	18.89b	2.71c	3477.60d
	K_60_	29.34ab	111.00a	16.73b	2.82b	4575.00c	27.67a	116.50a	19.08b	2.82bc	3760.53cd
	K_120_	31.17a	115.00a	18.09ab	2.89ab	5280.00ab	28.00a	118.50a	20.57ab	2.94ab	4096.64bc
	K_180_	30.50ab	119.00a	19.54a	2.99a	5746.67a	28.00a	122.50a	22.02a	2.99a	4575.13a
	K_240_	31.34a	112.33a	17.57ab	2.87ab	4931.67bc	27.84a	117.50a	19.23b	2.94ab	4221.49ab
ANOVA	K	***	***	***	***	***	***	ns	***	***	***
	V	***	**	***	ns	***	***	***	***	ns	***
	V×K	ns	***	ns	*	ns	ns	ns	*	ns	*
	Year	***	ns	***	ns	***					

Different letters within the same column indicate significant differences in response of the same cultivar to different treatments at 0.05 (*P*< 0.05); ns, not significant; *, **, and *** indicate significant differences at *P*< 0.05, *P*< 0.01, and *P*< 0.001, respectively. JG21, Jingu 21; ZZ10, Zhangza 10.

The results of variance analysis showed that there were significant differences in effective panicle number, grain weight per panicle, 1000-grain weight, and yield between Jingu 21 and Zhangza 10 with different potassium application rates, and the effect on spikelet number was inconsistent for the two years (**Table 4**). There was no significant difference in 1000-grain weight among the cultivars, but there were significant differences in effective panicle number, spikelet number, panicle grain weight, and yield. Appropriate potassium application resulted in a significant increase in the grain weight and 1000-grain weight of millet. Compared to that in the control, the average grain weight per spike of Jingu 21 was increased by 13.07% (2020) and 18.24% (2021), and the average grain weight per spike of Zhangza 10 was increased by 9.13% (2020) and 7.05% (2021). The average 1000-grain weight of Jingu 21 and Zhangza 10 increased by 7.14% and 5.43%, respectively. Excessive potassium application did not increase the yield composition. The yield in the two years was significantly different. Compared to that in 2020, the yield in 2021 was reduced by 27.18%–38.14% for Jingu 21, and 14.40%–22.41% for Zhangza 10. This can be explained from the perspective of rainfall and temperature during the millet growth period (**Figure 1**). During the grain filling period (from the end of July to the end of September), the rainfall increased, and the temperature decreased. This greatly impacted the grain-filling in 2021. Zhangza 10 matures early compared to Jingu 21; thus, the impact was smaller. Compared with 2020, the grain weight per panicle of Jingu 21 decreased, the grain-filling degree decreased, and the number of shriveled grains increased. The number of spikelets in Zhangza 10 increased, while the number of effective spikelets decreased.

### Stepwise regression analysis and path analysis of yield and carbon metabolism-related enzyme activities

The grain-filling stage is the critical period for millet yield formation, and carbon regulation is the essence of the source-sink relationship balance. To explore the effect of relevant factors (carbon metabolism-related enzyme activities between source and sink, and carbon metabolism-related enzymes in leaves and grains of millet at the grain filling stage) on yield, a stepwise regression analysis and path analysis was done. Leaf SPS (X_1_), leaf SuSy (X_2_), grain SPS (X_3_), grain SuSy (X_4_), leaf AI (X_5_), leaf NI (X_6_), grain AI (X_7_), and grain NI (X_8_) were set as independent variables, and millet yield (Y_1_: yield of Jingu 21; Y_2_: yield of Zhangza 10) was set as the dependent variable for the stepwise regression analysis. The following multilinear equations and fit values were obtained:


$$Y_{1}=5708.986+67.515\;X_{2}-67.252\;X_{4}-25.973\;X_{6}+19.050\;X_{7}$$

(R = 0.664, F = 15.333, P = 0.000, D = 1.347)


$$Y_{2}=1014.993+49.181\;X_{1}+44.127\;X_{2}\;+8.012\;X_{7}$$

(R = 0.807, F = 41.475, P = 0.000, D = 2.022)

It indicated that the above indexes were the main factors affecting the formation of millet yield. The yields of both cultivars are affected by leaf SuSy and grain AI. Path analysis further clarified the regulatory effects of the indices determined by stepwise regression analysis on the yield (**Table 5**). The results showed that leaf and grain SuSy had the greatest direct effect on the yield of Jingu 21. The leaf SuSy of Zhangza 10 plays a leading role in yield.

**Table 5:** Path coefficients of yield and sucrose metabolism-related enzymes.

Cultivar	Action factor	Correlation coefficient	Direct action	Indirect action					
				X_2_	X_4_		X_6_		X_7_
JG21	X_2_	0.314	0.603	—	–0.426		0.377		–0.240
	X_4_	–0.186	–0.611	0.420	—		0.303		–0.299
	X_6_	–0.416	–0.540	–0.421	0.343		—		0.202
	X_7_	0.363	0.505	–0.287	0.362		–0.216		—
				X_1_		X_2_		X_7_	
ZZ10	X_1_	0.586	0.404	—		0.274		–0.092	
	X_2_	0.842	0.630	0.176		—		0.036	
	X_7_	0.219	0.272	0.137		0.084		—	

JG21, Jingu 21; ZZ10, Zhangza 10.

## Discussion

### Potassium application improves photosynthetic carbon assimilation in millets

Photosynthesis is essential for numerous physiological processes that determine the growth and yield of millet. Rubisco and PEPC are critical enzymes for photosynthesis in C_4_ plants (such as millets). They determine the rate of carbon assimilation. Potassium deficiency reduces the photosynthetic rate and biomass in cotton; enzyme activity and content of Rubisco play an important role in this effect (Matsumura et al., 2020). Improved stomatal conductance, increased utilization of CO_2_ in the carbon assimilation process, and enhanced photosynthetic rate occur with an increase in potassium application rates (Lu et al., 2017). Here, with the increase of K content in leaves, *P*_n_, the activities of Rubisco and PEPC were significantly increased at the booting and filling stages, and there was a significant positive correlation between them. Potassium application increased photosynthetic carbon fixation efficiency by increasing the photosynthetic activity. FBPase is a key enzyme in the Calvin cycle and its activity is closely related to photosynthetic efficiency, photosynthate accumulation, and distribution. FBPase has a K^+^ binding site, and higher potassium fertilizer application rates significantly increase FBPase activity, which subsequently regulates the accumulation and distribution of carbohydrates and promotes yield (Hu et al., 2015).

### Effects of potassium application on millet photosynthetic carbohydrate metabolism enzymes activity

The leaf is the main organ for photosynthesis, and the assimilated products are transported to grains in the form of sucrose. The synthesis, transport, and accumulation of photosynthates in plants are regulated by the SuSy, SPS, AI, and NI. In C_4_ plants, SPS is mainly distributed in the mesophyll cells. SPS is a primary enzyme in sucrose synthesis, and its activity represents the sucrose transformation ability of flag leaf photosynthates (Binod et al., 2021; Hu et al., 2021). SuSy is a reversible enzyme with dual properties of sucrose synthesis and decomposition. The SuSy activity in grains regulates the distribution of carbohydrates in the source and sink organs (Fisher and Wang, 1995). The activities of SPS and SuSy in leaves are low in the early growth stage but increase at the functional transition stage (Wang et al., 2017). Potassium can regulate the activity of glucose metabolism enzymes, increase the reservoir strength, and promote assimilation and transport (Gerardeaux et al., 2010), as shown by several previous studies. Potassium fertilizer increases the activity of sucrose-degrading enzymes and decreases the SPS and SuSy activities at the sink, thereby preventing the resynthesis of decomposed sucrose, improving sink strength, and promoting the transport of photosynthates to the sink (Zhang et al., 2011). The sucrose degradation along with increased SuSy activity caused by potassium fertilizer helps improve sucrose metabolism and sink strength, resulting in the accumulation of soluble sugar at the sink (Wu et al., 2021). Liu et al. (2017) found that potassium application increases SPS activity in soybean seeds, decreases AI and NI activities, and increases sucrose concentration in seeds. Our study showed that sink organs increase the turgor pressure between source-sink organs and increase the SPS and SuSy activity in leaves at the filling stage. This is beneficial for increasing the supply of photosynthates at the source. Sucrose is transported to the sink (grains), where the high AI and NI activities promote sucrose hydrolysis, providing a substrate for the ADPG pyrophosphorylase-catalyzed starch synthesis. The higher activities of SuSy, AI, and NI in grains promote the continuous transport of photosynthetic products from source to sink under the action of turgor pressure differences (**Figure 4**). Furthermore, SPS and SuSy are primary enzymes mediating potassium sensitivity (Hu et al., 2015). Here, the activities of SPS and SuSy in different stages and parts of the plant showed that the variation range in Jingu 21 was greater than that in Zhangza 10 (**Figures 4A, B**). This might be one of the reasons for the different sensitivities of the cultivars to potassium. Potassium changes the balance of enzyme activities in sugar and starch metabolic processes; it increases the activities of AI, SuSy, SPS, and starch phosphorylase, and decreases the activity of ADPG pyrophosphorylase, thereby resulting in increased synthesis of hexose sugars, SuSy and ADPG pyrophosphorylase have the largest effect on enzyme activities in fruits (Luo et al., 2021). However, Gao et al. (2021) showed that potassium application significantly increases the activities of starch metabolism enzymes (such as sweet potato SuSy, and ADPG pyrophosphorylase) and starch synthases.

### Effects of potassium application on accumulation and distribution of carbohydrates in millet

Sucrose is the main photosynthate in plant transport, and starch is the primary energy storage material (Omondi et al., 2020). Grain-filling involves the translocation of photosynthates from the source (leaves, leaf sheaths, and stems) to the sink (panicle), and the accumulation of photosynthates eventually forms the yield (Wang and Zhang, 2020). As an essential nutrient element affecting the growth and development of crops, K fertilizer application has a significant effect on carbohydrate transport. Potassium deficiency decreases the sucrose conversion rate, which restricts the transfer of sucrose from para-leaf to cotton bolls, and the accumulation of sucrose in leaves leads to increased specific leaf weight or changes in carbohydrate structure (Hu et al., 2016). Hu et al. (2018) found that the soluble sugar and free amino acid contents of cotton leaves decreased with potassium application. The sucrose content of tomato leaves showed a significant increase with the increase in potassium application rates (Wang et al., 2020), while the sucrose content of cotton leaves decreased with an increase in potassium application (Hu et al., 2015). Here, with potassium application, soluble sugar and sucrose content decreased in leaves and increased in grains (**Figure 5**). High sucrose content in the grains provides sufficient substrate for starch synthesis, which benefits yield formation. The lower sucrose/starch ratio and the higher assimilate utilization capacity may be an important reason for higher grain yield in Jingu 21 compared to that in Zhangza 10 (**Figure 6**).

### Effects of potassium application on dry matter accumulation and yield of millet

Nutrient absorption is the physiological basis for dry matter accumulation; thus, optimizing the dry matter accumulation process is the basis for increasing crop yields (Nabin et al., 2022). Potassium application could effectively increase the K contents of each organ, and higher K applications have yielded higher proportions of stem distribution. However, despite the absorption of stem potassium, no significant changes in grain K contents have been observed (Ye et al., 2020). Potassium uptake by cereal crops mainly occurs before the flowering stage, and the K required during grain development is mainly obtained *via* transport from the vegetative organs (Zhang et al., 2016). Potassium deficiency restricts rice yield by affecting the potassium content, photosynthetic pigment content, and dry matter in various organs (Ranjan and Singh, 2021). Increasing potassium fertilizer rates could maintain the soil potassium balance, promote dry matter accumulation at the late growth stage, and increase the proportion of dry matter distribution in grains (Islam and Muttaleb, 2016; Hou et al., 2019). An increase in the potassium application rate resulted in improved degree of grain-filling and yield. However, excessive potassium application decreases yield (Qiu et al., 2014). In our study, potassium application significantly promoted K content and dry matter accumulation in the shoots of the two millet species but had no significant effect on the dry matter distribution ratio of the panicle. There was a significant difference in the yields of Jingu 21 and Zhangza 10 between the two years. Less rainfall during the entire growth period followed by higher rainfall during grain-filling period and concomitant drop in temperature affected grain-filling and resulted in a significant decrease in grain yield in 2021 (**Figure 1**). The optimal potassium application of Jingu 21 and Zhangza 10 were calculated using the linear plus platform model as 195.25–204.27 and 173.95–175.87 kg·K_2_O·hm^−2^, respectively (**Figure 7**).

Assimilates of carbon and nitrogen metabolism are the source materials for plant growth and development. Photosynthate regulation by carbon metabolism-related enzymes and their allocation between source and sink affect crop yield (Hu et al., 2022). Carbon metabolism is positively correlated with panicle number, panicle grain number, and 1000-grain weight (Wang et al., 2021). K^+^ is an activator for many enzymes and is closely related to enzyme activity. Leaf K content was positively correlated with SPS and SS, and was negatively correlated with AI and NI, which promoted the synthesis of leaf photosynthates and established a foundation for the yield. We further explored the regulatory effects of changes in carbon metabolism-related enzyme activities in the leaves and grains (during the grain-filling stage) of Jingu 21 and Zhangza 10 on yield. The yields of both cultivars were affected by leaf SuSy and grain AI activities. Jingu 21 yield was affected most by SuSy activity in both leaf and grain, which could optimize the sucrose synthesis rate and sink strength, and promote grain photosynthate accumulation under the effect of sink - source organ turgor difference. while leaf SS of Zhangza 10 played a dominant role in yield and promoted the generation of photosynthate at the source end, which laid a foundation for yield formation (**Table 5**).

## Conclusions

Potassium fertilizer application significantly: 1) increases the K content in every organ of the millet plant during the booting and filling stages; 2) enhances the activities of photosynthetic carbon assimilation enzymes as well as sucrose and starch metabolizing enzymes in leaves; 3) promotes carbon metabolism, and carbohydrate accumulation and distribution; 4) increases dry matter accumulation in shoots; 5) promotes carbohydrate transport from source organs to grains, and; 6) improves foxtail millet yield. Potassium promotes Jingu 21 yield mainly by coordinating the source-sink relationship between leaf and grain SuSy. It promotes Zhangza 10 yield by increasing the photosynthates production *via* leaf SuSy activity increase. The optimal potassium application of Jingu 21 and Zhangza 10 were calculated using the linear plus platform model as 195.25–204.27 and 173.95–175.87 kg·K_2_O·hm^−2^, respectively.

## Data availability statement

The original contributions presented in the study are included in the article/Supplementary Material. Further inquiries can be directed to the corresponding authors.

## Author contributions

YL performed the experiment and wrote the manuscript. MY revised the manuscript. LL modified the details. JZ assisted in the experiment. XY supervised the manuscript. YW provided the ideas and revised the manuscript. All authors contributed to the article and approved the submitted version.

## Funding

This work was supported by Research Program Sponsored by the State Key Laboratory of Integrative Sustainable Dryland Agriculture, Shanxi Agricultural University (No.202003-5); China Agriculture Research System of MOF and MARA (CARS-06-14.5-A28); Food System Research and Development Project in Shanxi Province, China (202001); Modern Millet Industry Technology System Project in Shanxi Province, China, and the program for the Scientific Research Foundation for the Returned Overseas Chinese Scholars, Shanxi Province(2020-068).

## Conflict of interest

The authors declare that the research was conducted in the absence of any commercial or financial relationships that could be construed as a potential conflict of interest.

## Publisher’s note

All claims expressed in this article are solely those of the authors and do not necessarily represent those of their affiliated organizations, or those of the publisher, the editors and the reviewers. Any product that may be evaluated in this article, or claim that may be made by its manufacturer, is not guaranteed or endorsed by the publisher.
